# Zebrafish Optokinetic Reflex: Minimal Reporting Guidelines and Recommendations

**DOI:** 10.3390/biology13010004

**Published:** 2023-12-20

**Authors:** Vanessa Rodwell, Manjiri Patil, Helen J. Kuht, Stephan C. F. Neuhauss, William H. J. Norton, Mervyn G. Thomas

**Affiliations:** 1 Ulverscroft Eye Unit, School of Psychology and Vision Sciences, University of Leicester, Leicester LE1 7RH, UK; 2 Department of Molecular Life Science, University of Zurich, 8006 Zurich, Switzerland; stephan.neuhauss@mls.uzh.ch; 3 Department of Genetics and Genome Biology, University of Leicester, Leicester LE1 7RH, UK; 4 Department of Ophthalmology, University Hospitals of Leicester NHS Trust, Leicester Royal Infirmary, Leicester LE1 5WW, UK; 5 Department of Clinical Genetics, University Hospitals of Leicester NHS Trust, Leicester Royal Infirmary, Leicester LE1 5WW, UK

**Keywords:** OKR, OKN, optokinetic, behavioural assay, zebrafish, danio rerio, guidance, guideline

## Abstract

**Simple Summary:**

Zebrafish form an ideal model for studying a wide range of ophthalmological and neurological conditions. The optokinetic reflex (OKR) assays in zebrafish models are proven to be a valuable tool for investigating these conditions. Despite its increasing popularity in recent years, the field lacks clear reporting guidelines for the assay. To better understand optimal reporting standards for an OKR assay in zebrafish, we performed a systematic literature review of 109 research papers exploring the animal, environmental, and technical factors that should be considered. In this article, we highlight multiple crucial factors, such as larval characteristics, sample size, fixing method, assay set-up, detailed stimulus parameters, eye recording, and eye movement analysis, necessary for preforming the assay. We have created the zebrafish optokinetic (ZOK) reflex minimal reporting guideline that will allow researchers to avoid future errors and create more reliable and transparent research.

**Abstract:**

Optokinetic reflex (OKR) assays in zebrafish models are a valuable tool for studying a diverse range of ophthalmological and neurological conditions. Despite its increasing popularity in recent years, there are no clear reporting guidelines for the assay. Following reporting guidelines in research enhances reproducibility, reduces bias, and mitigates underreporting and poor methodologies in published works. To better understand optimal reporting standards for an OKR assay in zebrafish, we performed a systematic literature review exploring the animal, environmental, and technical factors that should be considered. Using search criteria from three online databases, a total of 109 research papers were selected for review. Multiple crucial factors were identified, including larval characteristics, sample size, fixing method, OKR set-up, distance of stimulus, detailed stimulus parameters, eye recording, and eye movement analysis. The outcome of the literature analysis highlighted the insufficient information provided in past research papers and the lack of a systematic way to present the parameters related to each of the experimental factors. To circumvent any future errors and champion robust transparent research, we have created the zebrafish optokinetic (ZOK) reflex minimal reporting guideline.

## 1. Introduction

The optokinetic reflex (OKR) is a visual reflex that develops within the first six months in healthy children. Optokinetic nystagmus (OKN) is induced in response to a moving image, such as rotating black and white gratings, travelling across much of the visual field. Vertically oriented gratings would elicit a horizontal OKR whilst horizontally oriented gratings would elicit vertical OKR. OKR typically has two phases of eye movements. The “slow phase” equates to the movement of the eyes in the same direction as the moving stimulus to cancel out the retinal slip velocity. To reset the eyes, the slow tracking movement is routinely interrupted by a “fast phase” or “saccadic movement” in the opposite direction [1].

Abnormalities in OKN are a clinical finding in a variety of ophthalmic, vestibular, and neurological disorders. Examples of visual disorders associated with abnormal OKN include photoreceptor dystrophies, albinism, and *FRMD7*-related infantile nystagmus [2]. Neurological eye-tracking disorders can be divided into pursuit system disorders, such as floccular lesions, and quick phase disorders, such as progressive supranuclear palsy [3,4].

Common visual behavioural assays used in larval zebrafish include the optomotor response (OMR) assay and the OKR assay. In an OMR, fish reflexively swim in the direction of a moving stimulus. Due to the tracking of multiple fish simultaneously, an OMR is more advantageous for high-throughput screens [5]. In comparison, an OKR has the advantages of being more robust, reliable, and able to identify underlying diseases linked to the visual system and brain development. Reductions in visual acuity can be quantified using the OKR assay by altering spatial frequencies until no OKR is elicited [5]. Similarly, nystagmus has been reported in mutant zebrafish making it suitable for precisely measuring eye movements [6]. Due to their different merits, these assays are often used in tandem.

In addition to zebrafish, OKR has been successfully elicited in other animal research models, including rodents, rabbits, and cats [7]. Ethically, animal research must strive to follow the “three Rs” of animal research ethics: refine, reduce and replace [8]. Using zebrafish as an exemplar model aims to uphold these principles.

Firstly, zebrafish form ideal models for investigating OKN disorders, sharing an 87% disease gene homology with humans [9]. By 5 days post-fertilization (dpf), they already exhibit a fully functioning OKR, at which stage they are still legally classed as “unprotected organisms” in some countries, allowing for greater ease of research. Achieving successful OKR assays in zebrafish allows researchers to “replace” more sentient animals such as mice [8].

The establishment of clear reporting guidance aids in research design, the reproducibility of results, minimizing bias, and facilitating systematic review studies [10]. The National Institute of Health (NIH) propose that researchers have a scientific as well as a social responsibility to produce rigorous and transparent research through adherence to guidelines [10]. This allows each subsequent generation of researchers to verify a study’s validity, expand upon the current body of research, and further “refine” their method. Attaining high-quality results in animal models also provides valuable time-saving and economic benefits as well as more reliable results, which may translate to “reduced” numbers of animals per experiment [11]. 

The ARRIVE guidance, updated in 2020, is a well-known pre-clinical guidance for animal researchers which aims to standardise reporting practices across all published works [12]. It broadly outlines ten essential items including study design, sample size, outcome measures, and a description of experimental animals to provide a basic skeleton applicable to most animal research projects. Similarly, another paper called “the gold-standard checklist” approaches this from an animal ethics perspective and follows the ‘three Rs’ [11].

Though these guides provide a strong foundation for research design, neither are specific to a particular species. Guidance tailored to fish research does exist, including specific environmental and feeding steps that differ from other animals [13]. However, this guidance is dated and too generalizable, making it insufficient for reporting on complex research procedures such as OKR assays. 

More recent studies optimise experiments in fish models by summarising the literature and forming a standardised protocol [14]. Implementing this specific guidance on research methodology can further improve research rigour and quality and enhance the reproducibility of these procedures. Whilst in areas such as animal husbandry standardised protocols have been quick to emerge, only one exists for behavioural assays in larval zebrafish, which is the DAZL 2023 guidelines are for locomotion (OMR) assays for developmental neurotoxicity screening [15]. 

Recent reporting guidance has emerged on eye-tracking research methodology in humans but no such guidance currently exists for performing OKR behavioural assays in larval zebrafish [16].

In this paper, we aim to carry out a systematic review of different methodologies used to elicit, measure, and analyse the OKR in zebrafish. Based on the review, we developed the zebrafish optokinetic reflex minimum reporting guideline, identifying various factors in methodology that should be reported to ensure reproducibility and the correct interpretation of OKR results.

## 2. Materials and Methods

To perform a comprehensive literature review of current OKR reporting practices, we followed the PRISMA 2020 statement [17]. The search was conducted in May 2023 across three peer-reviewed literature platforms as follows: PubMed, Medline, and Science Direct. 

For the Medline and PubMed searches, we utilised the criteria as follows: “optokinetic” or “opto kinetic” or “OKR” and “zebrafish” or “danio rerio”. When conducting the search on the Science Direct database, we also included “zebrafish” or “danio rerio” in the keywords search bar.

### Inclusion and Exclusion Criteria

Papers were filtered for those in English and Italian to match the researchers’ language proficiency. 

Papers were only included if an OKR was performed as part of the study on larval zebrafish. Studies that used juvenile or adult zebrafish were excluded. Many studies failed to note the larval developmental stage in the abstract, hence the exclusion of “larvae” from our search criteria. Studies solely using OKR for neuro-imaging studies and not visual behaviour were excluded.

## 3. Systematic Review of Optokinetic Reflex Studies in Larval Zebrafish for the Basis for a Reporting Guideline

### 3.1. Search Results and PRISMA 2

**Figure 1:**
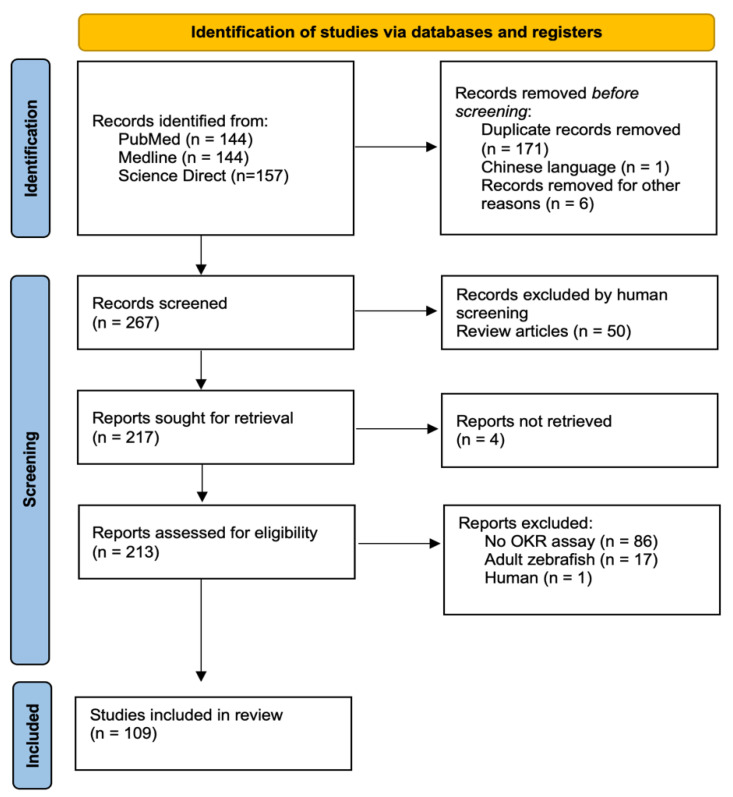
The PRISMA flowchart schematic detailing the review search and selection process adapted using PRISMA guidelines [17].

A total of 445 papers were identified across the three databases. Once duplicates and records were removed pre-screening, we had a total of 267 papers for screening. Finally, 109 relevant manuscripts were identified, with fifty of these being review papers (Figure 1).

**Figure 2:**
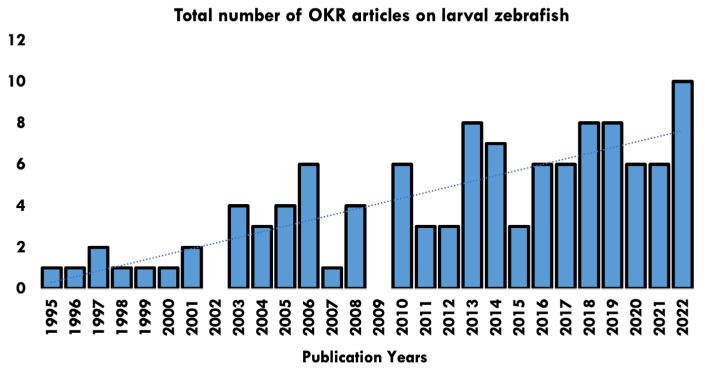
Publication years of papers identified in the OKR systematic review and number of articles published per year.

**Figure 3:**
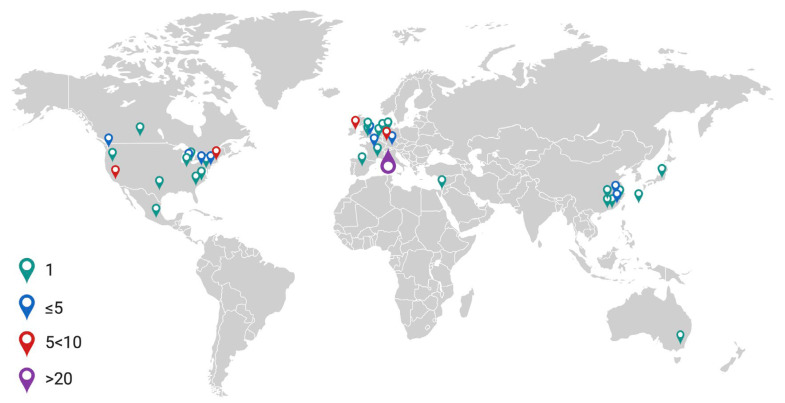
World map illustrating frequency of published articles in the literature search. Created using Biorender.com.

Papers ranged in date from 1995–2022, with a rising trend in the number of papers published each year (Figure 2). Studies originated across 40 research establishments worldwide (Figure 3). 

In this review, we have organised our results into two broad sections. The first section identifies the required parameters for designing an OKR assay and the second section covers current practices regarding the assay.

### 3.2. OKR Assay Parameters

#### 3.2.1. Larval Characteristics

##### Species of Fish

This review centres on zebrafish; however, it is important to note OKR assays used in other species such as goldfish and medaka.

##### Larval Developmental Stage

Days post-fertilisation (dpf) is a standard for reporting larval development. Zebrafish begin to develop an OKR at 3 dpf [18,19]. However, the developmental stage at which a mature OKR reflex can be elicited is debated, with some suggesting it is fully developed at 4 dpf [18] while others propose 5 dpf [20]. Physiologically, major ocular developmental milestones are met at 3 dpf, such as that of the retinal laminar structure, as well as the maturation of extraocular muscle function [21]. The focus in this review is on the larval stages; however, previous data suggest that visual acuity increases throughout the first year of development and then tails off at around 15 months of age [22].

#### 3.2.2. Immobilisation

To elicit an OKR, larvae must be immobilised. This is to prevent compensatory body movements from interfering with eye movement, allowing for clear visualisation under a dissection microscope [23]. For larvae at an early developmental stage, the chorion sac itself may be sufficient for immobilisation [19].

Three principal methods identified for immobilisation are using methylcellulose, agarose, and pins/needles.

##### Methylcellulose

Methylcellulose is a non-toxic, gelatinous, water-based compound which can preserve larval respiratory function. Larvae are embedded inside a Petri-dish and subsequently positioned using a dissecting needle or equivalent [24]. It is suggested to take ~1 min per larvae with some experience [25]. A concentration of 3% is of adequate viscosity to impede movement [26]. The higher the concentration of methylcellulose, the greater the risk of interference from eye movement [23].

##### Agarose

Agarose, a polysaccharide derived from seaweed agar, can be cooled to form a block. Larvae were embedded into an agarose block with eye and head cut-outs to allow for a full range of movement [24]. This procedure is considered more time-consuming compared with methylcellulose embedding; however, it removes all barriers between the stimulus and the zebrafish’s eyes [23].

##### Pins/Needles

In adult zebrafish behavioural assays, the fish is too large to remain immobile in methylcellulose. It can be mounted onto a silicon base surrounded by pins/needles to restrict movement. Zebrafish are still classed as larvae at ~20 dpf, at which point they are nearing their adult size and therefore could be pinned in a similar fashion [27,28].

#### 3.2.3. OKR Assay Set-Up

There are several different methods that have been developed for performing an OKR assay. This varies from a striped paper drum rotating around a larva to a stationary drum with projected stimuli and more modern LED arenas.

**Figure 4:**
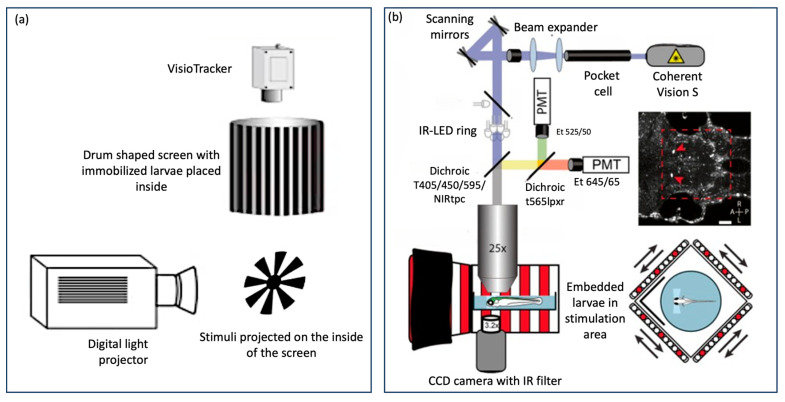
Automated “all in one” assays (**a**) VisioTracker schematic showing stimulus projector, rotating drum, and camera adapted from Video abstract in Mueller et al. 2011 [29] and (**b**) ZebEyeTrack inspired set-up adapted from Brysch et al. 2019 [30].

There are also automated “all-in-one” OKR assay technologies that have been used as a standard across many laboratories such as Visiotracker and ZebEyeTrack (Figure 4). These offer a combined tool for stimulus generation, eye movement recording, tracking, and analysis. Visiotracker involves the projection of stimuli onto a still paper drum [29]. ZebEyeTrack is a square LED arena [24].

##### Rotating Drum

**Figure 5:**
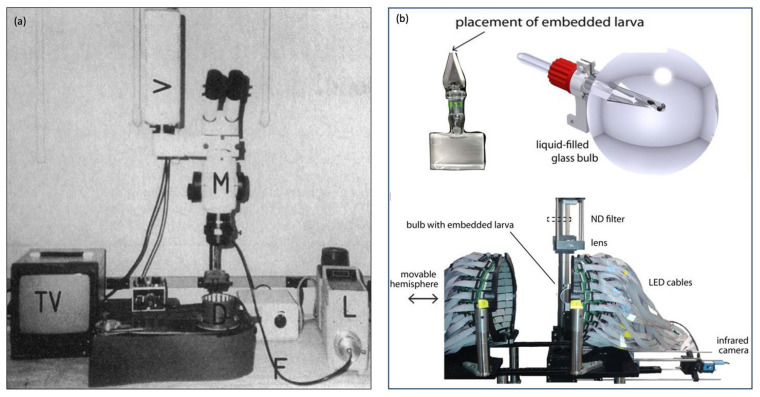
Different OKR set-ups (**a**) stimulus on rotating striped drum, figure adapted from Brockerhoff 1995 [20] and (**b**) Novel circular LED arena, figure adapted from Dehmelt 2021 [32].

This original technique for eliciting OKR utilised a striped paper drum which was rotated around larva by means of a belt and electrical motor (Figure 5a). These methods were pioneered in papers by Brockerhoff and Zou [20,31]. This technique is less common as more sophisticated methods of projecting stimuli have been developed.

##### Projected Stimulus

This method involved the projection of a moving computer-generated OKR stimulus. This allowed for more flexibility of the stimulation but has the increased complexity of projecting flat images onto a 3D drum or screen [29]. Distortions could be prevented through the use of a mirror or through software-based techniques [33].

The stimulus was most commonly projected onto a paper drum surrounding the larva [34] (Figure 4a) or using flat 2D screen made from diffusion film [35].

##### LED/LCD Arenas

This involves multiple screens arranged to form an arena (Figure 5b). The original arena that was used was square-shaped [24]. Physiologically, the larval visual field is 180 degrees. It is well understood that exposing larval zebrafish to full visual field stimuli will elicit a more robust OKR, hence novel spherical or cylindrical arenas have been constructed with larvae positioned centrally inside the tip of a narrow triangular glass stage [32,36].

#### 3.2.4. Optokinetic Stimulus

There is significant variation in the literature on reported stimulus characteristics and parameters. Computerised stimuli allow for greater flexibility in the range of stimuli provided [29].

##### Pattern

Vertical black and white square-wave gratings (alternating black and white bars/stripes) have been successfully used to elicit horizontal OKR in many experiments. Sinusoidal gratings have been used to evoke the natural environment which may allow for the smoother eye-tracking of stimulus [37]. Less commonly, a “random dot” pattern was used. This involves animated dots moving sinusoidally in opposite vertical directions generating a sensation of horizontal motion [38].

##### Stimulus Generation and Control

Traditionally, changing the stimulus on a rotating drum involves manually changing the gratings. Projection or LED assays allow for computer-generation and control of stimuli parameters, such as contrast and speed, during experiments in real-time [29]. Many different types of software can be used.

##### Frequency and Velocity

**Figure 6:**
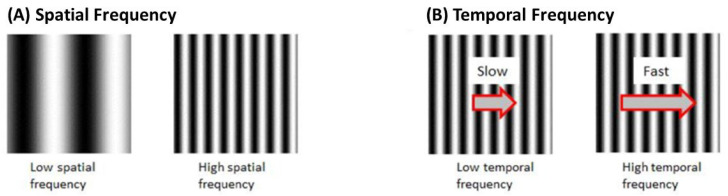
(**A**) Spatial frequency of OKR stimulus is determined based on the number of black and white gratings (**B**)Temporal frequency is based on the number of cycles per unit time.

Each set of black and white gratings is known as one cycle. Spatial Frequency (SF) is the number of cycles per unit distance. A higher SF indicates a higher number of gratings with a smaller width. Velocity is the rate of stimulus rotation. Temporal frequency (TF) is the number of cycles per unit time. TF is the product of SF and velocity (Figure 6).

OKR gain is dependent on SF and velocity. Research shows that the stimulus is no longer detected through raising SF and velocity to the limit of larval vision, thus gain decreases [33]. The highest spatial frequency that an eye can still resolve has been described as the “optical cut-off frequency” [39].

On traditional drums, SF could be substituted for manual measurements of the drum. Computer-generated stimuli allow for accurate calculation of spatial frequencies.

##### Contrast

The stimulus contrast is the light to dark ratio of stimulus gratings. Research on contrast sensitivity found gain increased by 0.77 per log unit contrast [33]. A high contrast is thus recommended to evoke strong OKR in zebrafish. 

##### Distance of Stimulus from Larval Eye

The distance from stimulus to larva impacts all the above parameters. Optimisation experiments can be performed by varying the stimulus distance while maintaining the same spatial frequency [39]. The methodology used to measure the distance of stimuli varied. Some calculated the radius of the rotating drum, assuming the larva was centrally located. Others took more precise measurements of the distance between the larval eye and stimuli [40].

##### Direction of Rotation

Altering the direction of rotation heightens the robustness of the OKR [41]. Frequent rapid changes in direction have been used to reduce the number of quick phases and make it easier to calculate slow phase velocity [42]. 

The direction of optokinetic stimulus rotation affects the intensity of the OKR elicited on monocular stimulation of lateral-eyed vertebrates, including zebrafish. A naso-temporal stimulus will elicit a greater response than a temporo-nasal stimulus [43]. This is due to the characteristic asymmetry of the OKR in temporal-to-nasal (T-N) direction under monocular stimulation.

##### Monocular vs. Binocular Stimulation

Traditionally, larvae were placed inside a rotating drum and binocularly stimulated. However, the OKR in the eye of interest can be affected by contralateral eye stimulation [32]. There are various methods for achieving monocular stimulation, such as positioning the larva to be perpendicular to the stimulus or isolating half the visual field with a barrier. Stationary gratings were said to be more effective than black, white, or aluminium shields at isolating one eye from receiving any stimulation [32].

##### Light Intensity

Initial development of zebrafish retinomotor movements at 5 dpf allows for functional light adaptation [6,44].

When considering “light intensity”, this often refers to the light emanating from the assay itself, although impact of the lighting in the surrounding environment should not be neglected. 

There are many different units for light intensity. Lumen (lm) is the total quantity of visible light emitted from a source in any direction. Illumination ‘Lux’ (lx) is the measure of light intensity received on a surface that is being lit. Candela (cd) is the light intensity emitted from a source in a certain direction. Luminance (Nit, cd/m^2^ or Ft/L) is the light intensity emitted from a certain surface area (for example a computer screen) in a certain direction. It has been reported that when varying light intensity above 3.2 cd/m^2^, OKR gain was not impacted. However, gain dropped significantly below 0.36 cd/m^2^, indicating that zebrafish struggle to see stimuli with very low illumination [33]. Illumination at the position of the larval eye has been measured using a photometer positioned at the centre point of the assay [37,45].

#### 3.2.5. Experiment

##### Throughput and Duration

The experimental throughput is the number of larvae one can measure in a given time which will determine the duration of the experimental phase of research. High throughput has been achieved through simplifying the OKR set-up, the automation of OKR analysis, or the use of commercially available systems like Visiotracker and ZebEye track [24,29]. When manually recording OKR, the throughput of an OKR assay was limited to one. When measuring one larva at a time, a throughput of ~600 larvae in a single day (10 h) was reported [25]. On a grander scale, others have managed a throughput of ~77,000 larvae over three years [34].

Eye movements of only one larva can be viewed at a time. Modern techniques combining video recordings with new eye-tracking software enable multiple simultaneous measurements of OKR [24].

##### Sample Size

Zebrafish studies vary immensely in the total sample size of larvae utilised for an experiment. Genetic screens can generate large sample sizes [34]. However studies trialling new OKR methods have comparatively smaller sample size [29]. In the context of a forward genetic screen for recessive mutations, it is often advocated that screening 12 fish from a single clutch can significantly increase the likelihood of detecting a genetic mutant [26]. Additionally, assessing whether fish exhibit no OKR responses can be particularly insightful for genetic screening or toxicology assays. However, it is important to note that such assessments should be tailored to the specific objectives of the experiment, taking into account the desired outcome measures and the necessary sample size calculations [26].

##### Time of Day

Zebrafish, like humans, possess a natural circadian rhythm which influences their behaviour. Thus, performing behavioural assays at different times of the day may skew results [25]. Overnight, we know zebrafish photoreceptors are non-active due to the dismantling of ribbon synapses, meaning they do not naturally respond to any visual stimuli [46]. More importantly, throughout the day their biological rhythms have been found to cause nuanced fluctuations in visual sensitivity [47]. There is evidence that circadian regulation weakly affects the OKR by influencing the visual transduction cascade [48].

Furthermore, when dealing with larvae 2–5 days post fertilisation, recordings ±8 h apart will affect the maturity of the OKR, as mentioned in the section of larval characteristics of OKR assay parameters.

#### 3.2.6. Recording of Eye Motion

##### Manual

Traditionally, papers recorded changes in eye motion manually through a dissection microscope [20].

#### 3.2.7. Cameras

Using cameras to record eye movements provides more flexibility. Recordings can provide rapid automated eye movement analysis in real-time and an accurate calculation of gain by measuring eye velocity [24]. Alternatively, videos can be used for manual analysis [49]. The industry standard camera is the CCD (charge-coupled device) which transmits data through analogue signals. A CMOS (complementary metal-oxide semiconductor) camera is a more modern cost-effective device which transmits data through digital signals. The quality of new-generation CMOS cameras is comparable to a CCD.

#### 3.2.8. Analysis of Eye Motion

##### Manual vs. Software

Historically, manual screening has been favoured in OKR assays, particularly for its perceived ability to reduce false positives [25]. However, an often overlooked aspect in these studies is the implementation of double-blind methodologies. Such approaches, while instrumental in reducing observer bias, especially in manual evaluations, are not commonly reported. Furthermore, their applicability can be limited in contexts where fish display distinct phenotypes, such as hypopigmentation, which can inadvertently influence the observer’s assessment. Despite the potential benefits, it is important to note that manual methods also pose a risk of increased bias and human error [33].

In contrast, the emergence of eye-tracking software has provided an alternative that offers both rapidity and accuracy. Some of these software solutions utilise proprietary codes not openly shared with the public, while others are based on open-source environments like Python or MATLAB, offering broader accessibility and customisation options [1]. Advanced all-in-one assay models, such as Visiotracker and ZebEyeTrack, integrate eye-movement analysis software, further streamlining the OKR assessment process (Figure 4).

The choice between manual and software-based analysis in OKR assays should consider these factors, with an emphasis on minimizing bias and enhancing the reliability of results. Future studies employing manual screening are encouraged to explore and report the use of double-blind procedures where applicable to enhance the objectivity of their findings.

##### Quantifying the OKR Assay

Generally, a “positive OKR” outcome is defined as the presence of at least one slow phase or quick phase [18]. Some require both to be present for a positive response [50]. OKR outcomes can also be quantified. Quick phase/saccades can be counted or provided as a frequency [51,52].

Using software for eye-tracking, slow-phase velocity can be used to determine gain, a well-known indicator of visual impairment [53]. Gain is calculated as the ratio of eye velocity to stimulus velocity. Alternatively, an index for rating OKR outcomes on a custom-made scale can be devised [34,54,55].

## 4. Overview of Parameters Utilised in Designing the OKR Assay

The literature review highlights various factors reported when planning and conducting an OKR assay in zebrafish. We have explored how these factors (developmental stage of larvae, immobilisation method, assay set-up, pattern, stimulus parameters and presentation of stimulus) could influence the OKR assay results and its interpretation. 

Key methodological considerations reported to elicit a robust OKR response involve selecting larvae of 5 dpf or above and using an assay set-up with full-field monocular stimulation. Strong evidence indicates selection of the stimulus spatial frequency and velocity is important for OKR outcome, with attention required to not surpass the “optical cut-off frequency” which results in poor OKR responses due to the inability of the fish to resolve the stimulus. A higher stimulus contrast will also produce a better response. Once robust OKR responses have been elicited, the final data quality is further dependent on eye movement recording and analysis. Utilising appropriate software-based approaches instead of relying on manual methods can greatly enhance the automation of eye movement measurements and effectively mitigate observer bias.

### 4.1. Existing Reporting Practices

The above-mentioned variables directly or indirectly impact on OKR experimental design, analysis, and interpretation. From the 109 relevant studies identified, we calculate the percentage of papers which contain the appropriate details for each variable. We will go on to discuss the minimal reporting guidance considering the literature. 

#### 4.1.1. Larval Characteristics

##### Species of Fish

A total of 100% of papers referenced the species either as ‘zebrafish’ or ‘Danio rerio’.

##### Larval Developmental Stage

**Figure 7:**
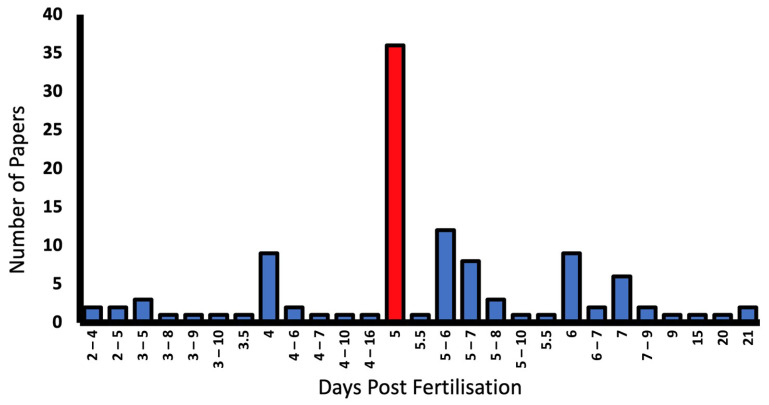
Larval developmental stage (days post fertilisation (dpf)), and number of reporting papers in the literature. The most common larval stage used in OKR experiments was 5 dpf (red bar).

Only 7.3% of studies failed to report the developmental stage of larvae [34,35,56,57,58,59,60]. Larvae from 2–21 dpf were used, with 5 dpf being most commonly reported in 35.6% of papers. Some report dpf as a range as outlined in the graph (Figure 7). 

#### 4.1.2. Immobilisation

The immobilisation method was detailed in 88% of studies, with a further 6.4% referencing past methodology [57,61,62]. 

**Figure 8:**
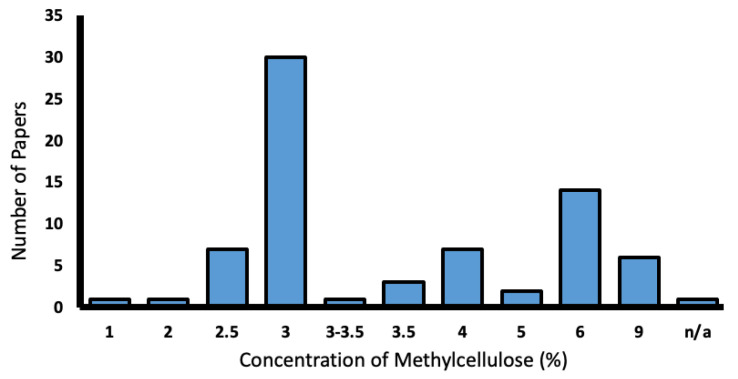
Range of methylcellulose concentrations used in the literature (%).

From this total of 103 papers, methylcellulose was the most popular mounting medium and was utilised in 70.9% of papers. Concentrations of methylcellulose varied from 2.5–9%, with 3% being the most common (Figure 8). 

**Figure 9:**
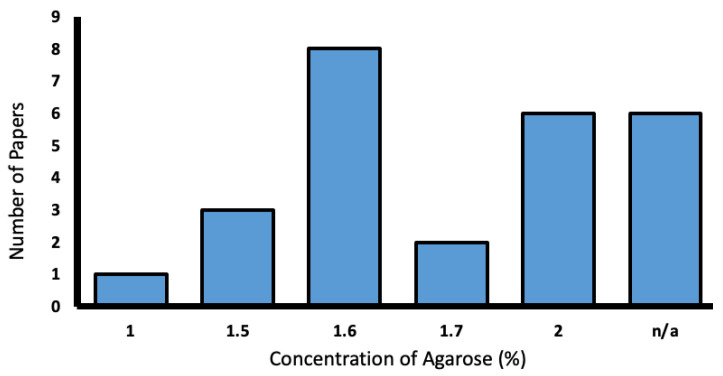
Range of low-melting agarose concentrations used in the literature (%).

Agarose was used in 25.2% of the reporting studies. Concentrations varied from 1–2%, with most favoring 1.6% (Figure 9).

Pins/needles were used in 1.9% papers, where the larvae exceeded 20 dpf [63]. A further 1.9% reported no immobilisation method due to larvae being inside the chorion. 

#### 4.1.3. OKR Assay Set-Up

The OKR assay set-up was detailed in 89.9% of studies, with a further 5.5% referencing another paper’s methodology. A total of 3.7% do not mention OKR methodology [58,64,65,66].

Popularity of each method:

A. In 39.4% of papers, the OKR assay used a standard rotating drum.
B. The OKR stimulus was projected onto a stationary surface in 43.1% of papers.
C. The LED/LCD arena method was used in 13.8% of papers.

#### 4.1.4. Optokinetic Stimulus

##### Patterns

In 86.2% of papers the pattern of the stimulus was mentioned. A total of 55% of manuscripts utilised a standard stimulus of black and white stripes/gratings. A total of 4.7% of papers did not report their stimulus, but gratings were present in images of the OKR set-up. In 23.8% of papers the stimulus was sinusoidal and 0.03% used random dot patterns [38,67]. Seven papers with no pattern used Visiotracker software and thus likely used their standard gratings. 

##### Stimulus Generation and Control

From 60 manuscripts with projected or LED-based stimuli, 66.7% referenced software for stimulus generation, with 22.5% of these opting for LabVIEW (National Instruments) [68,69,70], whilst 12.5% used MATLAB [1,32]. The rest used other software: Image-J, Simple DirectMedia Layer, PsychoPy, NIH Object Image and Python Library Vision (Table 1). This is excluding papers known to use Visiotracker or Zebeyetrack software.

##### Stimulus Parameters: Frequency and Velocity

A total of 81.7% of papers included a value for SF or a corresponding parameter. A total of 84.4% of papers provided a value for the stimulus velocity/temporal frequency. They have been divided based on the OKR method (Table 2).

For papers using the rotating drum method, 90.7% reported stimulus parameters. Of these, 84.6% reported the SF as degrees of a cycle (ranging from 9–40 degrees), with 18 degrees as the most popular. The TF was provided as the number of rotations per minute in 69.2% of papers (ranging from 3–20 rpm), with 6–8 rpm being the most common. 

From all methods using a projected stimulus, 91.5% provided some stimulus parameters. Of these, 55.8% recorded SF in cycles per degree (cpd) (from 0.01–0.6 cpd). The velocity in degrees per second (from 2.7–30 deg/s) was noted in 81.4%. Median values of 0.06 cpd and 7.5 deg/s were most common [33].

For LED assays, 73.3% of papers reported stimulus parameters. Notably, all of these papers also reported velocity in degrees per second (from 5–48 deg/s). SF was mostly reported in cycles per degree (from 0.033–0.066 cpd), with one paper using a random pattern omitting a SF [81]. The most commonly used settings were 0.033 cpd with 10–15 deg/s [36].

##### Stimulus Duration 

The time of exposure to a stimulus was given or referenced in 67% of papers. This varied greatly from just 3 s [33] to as long as 20 min [81]. Over a third of these papers used a timing of 1 min [41,51,54,84,85]. A duration of 2 min was also common [52,86,87]. 

##### Direction of Rotation

72.5% of manuscripts highlighted the direction of stimulus rotation. A bidirectional stimulus was used or referenced in 65.1% of papers [79,88]. 

#### 4.1.5. Lighting

##### Distance of Stimulus from Larval Eye

Authors provided the distance between stimulus and larva in 26.6% of papers. Of these, 51.7% provided a drum diameter and 34.5% noted the specific measurement from the larval eye to the stimulus, from 6 cm to 22 mm (Table 3).

A further 13.8% of the papers provided a complex 3D location with more modern circular LED arenas.

##### Monocular vs. Binocular Stimulation

20.2% of manuscripts stimulated larvae monocularly [38,81,88,93]. Most papers, 48.6%, did not specify whether the stimulus was monocular or binocular.

##### Contrast

Contrast was mentioned in 31.1% of studies. One third of these studies, measuring contrast sensitivity, provided a broad range of contrast levels tested from ~1–5% to 100% contrast [6,44,79], 50–100% [55], and another from 20–99% [71]. The rest of these studies used a fixed high constant contrast of 85–100% [88,93,94]. 

In studies that did not mention contrast, light parameters in the forms of intensity or luminance were stated in 14.7% of papers. Only 11% mentioned both contrast and lighting parameters (Table 4).

#### 4.1.6. Experiment

##### Throughput

Only one trial using ZebEyeTrack reported on the assay throughput accuracy. A high OKR accuracy was found when measuring six zebrafish larvae simultaneously, comparable to individual stimulation [24]. 

##### Sample Size

A total of 84.4% of publications mentioned the sample size of larvae. One paper measured OKR from over 70,000 larvae over a three-year time period [34]. Most studies reported an average sample of 6–15 per larval group.

##### OKR Time of Day

Only 14.7% of manuscripts include time parameters for performing an OKR. Just over half of these had broad parameters spanning morning and afternoon, between 8 am and 7 pm. The most common time of day was between 12 and 6 pm, with 37.5% of these papers performing an OKR exclusively in the afternoon [53,101,102].

##### Recording of Eye Motion

64.2% of researchers used cameras for recording eye movement. Of these, 55.7% used CCD cameras [103], with 8.6% utilising newer CMOS technology [67]. A total of 44.2% were stated to be infrared sensitive.

##### Analysis of Eye Motion

Analysis software was used in 54.1% of papers (Table 5). A total of 35.5% of these used LabView. 

Eye movement analysis tools were not used in 45.9% of publications. Only 16.6% of these papers explicitly stated the analysis was done manually [104].

**Table 5:** Number of papers that reference eye movement analysis software.

Analysis Software	Number of Papers	Paper Reference
LabView	16	[6,33,39,42,44,55,68,69,71,75,82,83,90,91,103,105]
LabView/Matlab	4	[73,93,106,107]
Labview/Tracker 4.0	1	[72]
LabView/ImageJ	1	[88]
Cell F	1	[88,108]
Viewpoint OKR	2	[61,84]
Matlab	9	[1,40,56,65,70,78,86,89,109]
ZebEyeTrack (Matlab)	7	[24,30,32,65,110,111,112]
AmScope/Matlab	1	[113]
Python/Matlab	1	[67]
Bonzai/Matlab	1	[94]
Proprietary	1	[29]
ScanImage	1	[77]
Media Cybernetics IPP6	1	[114]
Image J	4	[34,38,59,100]
Object Image2	1	[99,108]

Movement analysis software.

##### Quantifying OKR Assay

Authors reported on how they quantified the OKR in 89.9% of publications. From these, the most common outcomes measured were eye velocity/gain and saccade rate in 51% and 28.6% of papers, respectively. The number of positive OKR responses or ‘saccades’ was reported in 33.7% of papers (Table 6).

**Figure 10:**
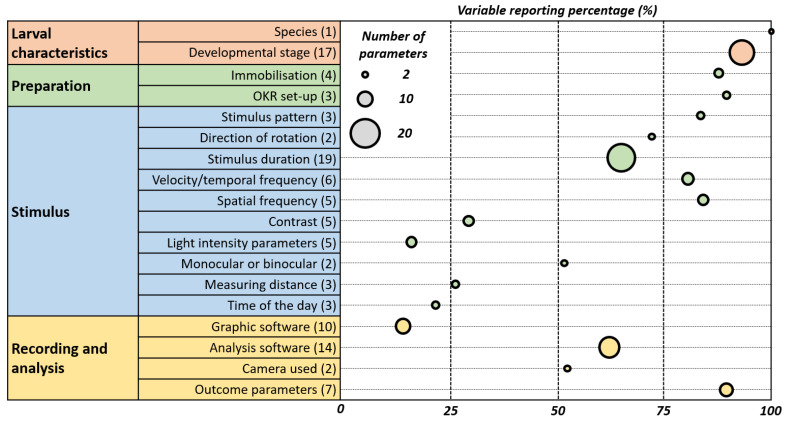
Weighted Bubble Chart Illustrating Reporting Variability and Prevalence in OKR Studies. This chart depicts the reporting practices in optokinetic reflex (OKR) studies. Each bubble represents an OKR parameter, with its position on the *x*-axis indicating the percentage of papers reporting it. The size of the bubble reflects the variability in how the parameter is reported across studies—larger bubbles denote greater variability. This visualisation highlights the prevalence and consistency of reporting for each parameter, underscoring areas where standardised practices may be needed.

The inconsistencies in OKR assay reporting are shown in Figure 10 which emphasizes the need for its standardization.

## 5. ZOK Reporting Guideline for Optokinetic Reflex Studies in Larval Zebrafish

Based on our systematic literature review, there is significant variability in the methodologies employed and the level of detail reported for OKR assays in larval zebrafish. New researchers require more support to fully take advantage of the OKR assay. Hence, the following minimal reporting guideline has been devised to aid future researchers (Table 7). As a primary step, the provided guidance table offers a structured outline of the necessary procedures. This entails the researcher to make the choice of an appropriate OKR assay method from (a) a rotating drum, (b) a projection, or (c) an LED arena. When uncertainty arises in the selection of parameter values for each method, we suggest referring to the section of existing reporting practices of this review.

## 6. Discussion

After reviewing the existing literature, it is clear that several factors impact measurements reported from OKR assays, including larval characteristics, immobilization methods, assay set-ups, stimulus parameters, and analysis techniques. Our systematic review of 109 papers highlights the prevalent issue of insufficiently reported variables, possibly due to lab-specific approaches and a lack of standardized reporting guidelines. We propose using a simple minimal reporting guidance table (Table 7) which serves as both a checklist for researchers establishing an OKR assay and a tool for peer reviewers assessing a study. Whilst different laboratories may utilise various ranges of parameters, the accuracy of OKR assays can still be achieved. The proposed reporting guidelines are designed to foster reproducibility and transparency rather than dictate a uniform experimental approach. This flexibility is particularly crucial when working with a spectrum of phenotypes in behavioural research. Our goal is to support a broad application of these guidelines which are adaptable to diverse research settings and methodologies.

With the advent of CRISPR-Cas9 mutagenesis techniques, the ease of modeling human mutations in zebrafish has significantly increased [116,117]. Through integrating these advances into genetic engineering with state-of-the-art visual behavioural assays, such as the OKR, researchers are not only able to model diseases linked to retinal development [118,119,120,121,122,123] but also explore cis-regulatory variants affecting neurological development or retinal function [124,125]. This approach has shown effectiveness in modeling infantile nystagmus [6], examining its impact on OKR development, and evaluating therapeutic responses analogous to those used in human treatments [75]. Such studies underscore the potential of using zebrafish as a versatile model for understanding and treating human visual disorders and, importantly, the role of well-designed OKR assays to objectively determine disease status and therapeutic responses.

Certain challenges were encountered during the execution of this review. Determining the influence of variables on the zebrafish OKR proved challenging due to limited existing research in the field. A potential weakness was that nine assay methodology papers were included in the review which conducted no OKR investigations other than displaying the set-up of their equipment. This could have slightly negatively affected the percentages concerning reporting practices. However, these papers were included in the review because assays were carried out on wildtype zebrafish. Employing better reporting in methodology papers can also be advantageous in proving that a proposed method is effective.

It was also difficult to identify the optimal parameter for a variable where values differed greatly across the studies. The modal “ideal” value we calculated may be subject to sampling bias as larger laboratories publishing multiple papers use the same protocol, potentially influencing these results. However, it is noteworthy that these high frequency publications prove their OKR assay protocol to be highly effective and perhaps it is a good opportunity for others to adopt these practices. This review provides great transparency for researchers through its systematic tables with references for various parameters. Additionally, legal restrictions in some countries probably influenced the popularity of using 5 dpf larvae. It is important for new researchers to consider that older larvae might exhibit a stronger OKR response. While our paper focused on larval OKR studies, our examination of studies involving adult zebrafish indicates that, despite marked differences in experimental procedures such as age and immobilisation methods, the fundamental reporting parameters are similar. Consequently, we propose that our reporting guidelines can also be relevant and beneficial for studies involving adult zebrafish.

Finally, the OKR assay has been found to be a popular and accurate tool for visual system analyses in larval zebrafish. Our goal is to establish ZOK as a baseline standard that enhances research transparency while remaining adaptable to individual research needs. Notably, this guidance is flexible and accommodates various assay configurations. We anticipate the use of the ZOK reporting guideline can lead to higher quality research being performed on zebrafish models.

## Figures and Tables

**Table 1:** Number of papers that reference each software for stimulus control.

Software	Number of Papers	Reference
Labview NI	9	[42,65,68,69,70,71,72,73,74]
Custom NI	1	[75]
Image J	1	[38]
Simple direct media layer	2	[41,44]
PychoPy	2	[76,77]
Matlab	5	[1,32,35,67,78]
Matlab+ Pychotoolbox	2	[79,80]
Microsoft windows	1	[33]
Python library vision	2	[81,82]
Vision egg	1	[83]

**Table 2:** Frequency of various stimulus rotation parameters reported in the literature.

Method	Stimulus Rotation Parameters	Number of Papers
Rotating Drum	Degrees + rpm ^1^	23
	Width of stripes + rpm	6
	Width of stripes + Hz	1
	Degrees + deg/s	7
	Degrees + deg/s + Hz	2
	Number of stripes + rpm	2
	Cpd ^2^ + rpm	1
	Number of stripes/s + deg/s	1
Projection	cpd + deg/s	23
	cpd + deg/s + Hz	2
	Deg/360 cycle + Deg/s	4
	Width of stripes + Deg/s	2
	Width of stripes	1
	Width of stripes + Hz	1
	Deg + deg/s	1
	cpd + Cycles/min	1
	Deg + Hz + Deg/sec	1
	Number of stripes per cycle + rpm	1
	Width of stripes + mm/s	1
LED assay	cpd + deg/s	4
	cpd + deg/s + cycles/sec	1
	cpd + deg/s + cycles/360 deg	2
	cycles/s + cycles/360 deg + deg/s	1
	Cycles/sec	1
	cpd	1
	Deg/s	1

^1^ Rotations per minute and ^2^ Cycles per degree.

**Table 3:** Distances measured from larval eye to stimulus (cm).

Distance (cm)	Number of Papers	Reference
0.22	1	[89]
0.33, 0.62, 0.45 ^1^	1	[39]
2	2	[35,40]
4.65	5	[6,74,90,91,92]
6	1	[71]

^1^ Three distances trialled in this paper.

**Table 4:** Number of papers that report light parameters in the literature.

Light Intensity	Number of Papers	Reference
Light intensity W/cm^2^	4	[28,50,86,95]
W/cm^2^ and Log	4	[41,96,97,98]
Log	2	[20,27]
luminance cd/m^2^	1	[32,99]
Luminance fL	2	[18,19]
contrast % and luminance cd/m^2^	5	[6,33,71,91,100]
contrast% and max illumination lux	6	[42,70,75,88,93,94]

**Table 6:** Number of papers outcomes for reporting the OKR.

Outcome	Number of Papers	Example References
Saccade rate	28	[51,104,111]
Number of saccades	11	[87,94,113]
Average eye velocity/gain	50	[44,90,92]
Positive OKR	22	[25,99,115]
Eye movement frequency	2	[84,114]
Eye movements 1–20 scale	2	[54,55]
Average motion	1	[67]

**Table 7:** The ZOK recommended minimal reporting guidelines.

Section	Aspect	Examples
Larval characteristics	Species and Strain	Zebrafish/Danio rerio (AB WT)
	Stage of development	Days post fertilisation
Preparation of larva	Immobilisation technique	Methylcellulose/agarose/pins
	Concentration of solution	Concentration (%)
	Container and size	35 mm petri dish, 21 mm tube
	Position of larva	Facing outwards dorsal side up
OKR Assay method	Manufacturer/Model	Visiotracker, ZebEyeTrack, Custom
	Set-up	Drum/projection/LED
Rotating Drum	Drum material	White paper/Cardboard
	Rotation method	Rotating platform/belt and motor
	Diameter	(cm)
Projection	Shape of screen	Drum/Semicircular screen
	Screen material	White paper/diffusion film
	Projection technique	LCD projector
LCD/LED arena	Shape of arena	Square/circular
	Number of LCD/LED panels	4 LED panels
	Arrangement of panels	Bonded at right angles
Stimulus	Stimulus pattern	Gratings/sinusoidal/random dot
	Direction(s) of stimulus rotation	CW/CW + ACW *
	Stimulus Duration	(s)
	Contrast	(%)
	Eyes stimulated	Monocular/Binocular
	Distance from larval eye	(cm)
	Light Intensity	(W/cm^2^)
Rotating Drum	Degrees of cycle	(deg)
	Velocity	(rpm) and (deg/s)
ProjectionLED arena	Wavelength	IR (nm)
	Spatial frequency	Cycles per degree (cpd)
	Velocity	(deg/s)
	Graphics software	Matlab/Labview
Experiment	Trials	Number
	Assay Throughput	(*n* = 1)
	Sample size	Total larvae (*n* = 40)
	Time of day	2–7PM
Recording of eye rotation	Camera	CCD/CMOS
	Dissection microscope	Name
Analysis of eye rotation	Software	Matlab/Labview
	Outcome	Eye velocity/Saccade rate/Gain

* Clockwise and anti-clockwise.

## Data Availability

All data presented is available within the manuscript and references.
